# Plant Disease Suppressiveness Enhancement via Soil Health Management

**DOI:** 10.3390/biology14080924

**Published:** 2025-07-23

**Authors:** Chinmayee Priyadarshini, Rattan Lal, Pu Yuan, Wenshan Liu, Ashna Adhikari, Santosh Bhandari, Ye Xia

**Affiliations:** 1 Rattan Lal Center for Carbon Management and Sequestration, School of Environment and Natural Resources, College of Food, Agricultural and Environmental Sciences, The Ohio State University, 2021 Coffey Rd, Columbus, OH 43210, USA; priyadarshini.6@buckeyemail.osu.edu; 2 Plant Pathology Department, College of Food, Agricultural and Environmental Sciences, The Ohio State University, 2021 Coffey Rd, Columbus, OH 43210, USA; yuan.1282@osu.edu (P.Y.); liu.11241@osu.edu (W.L.); adhikari.168@buckeyemail.osu.edu (A.A.); bhandari.125@buckeyemail.osu.edu (S.B.)

**Keywords:** disease-suppressive soil, pathogen and disease control, sustainable agriculture, soil-borne pathogens, carbon pool, nitrogen cycle, environmental factors, beneficial microorganisms, soil health, best management practices

## Abstract

Managing soil-borne pathogens and diseases in plants is particularly challenging because the pathogens that cause them can persist in the soil for extended periods, often resulting in repeated crop damage in affected areas. These destructive diseases compromise plant health by weakening the root systems, which makes the plants more susceptible to environmental stress and nutritional deficiencies. Every year in the United States, a whopping $9.6 million is allocated to reverse the harmful effects of pesticides on humans, plants, animals, and the environment. On the contrary, disease-suppressive soils offer an effective strategy for controlling pathogens while ensuring the least contamination of the environment. These soils can be managed by both conventional and advanced methods, such as reduced tillage, crop rotation, organic amendments, nanoparticles, omics approaches, and biofumigation. However, these soils can be local in nature, and their properties might be disrupted by common agricultural practices like tillage and agro-chemical application. This review synthesizes the concepts and mechanisms of disease suppression in soils and explores the ways that can be improved through the management of soil health for enhanced plant health and yield.

## Simple Summary

This review article discusses the importance of disease-suppressive soils in the management of both soil and plant health. It explores the concept of disease suppressiveness, its emergence in history, its types and mechanisms, and the pivotal role played by soil properties and overall soil health in determining the efficiency of disease-suppressive soils. The article further reviews the latest research trends in managing and enhancing the effectiveness of these types of soils and canvases their future uses.

## 1. Introduction

Soil-borne plant diseases are caused by various types of pathogens, such as fungi, bacteria, and nematodes, which infect the plants through their roots. These pathogens cause severe damage, including root rot, wilting, and stunting, significantly reducing crop yield and quality [1]. Soil-borne pathogens and diseases disrupt the health of the plants by weakening root systems, making the plants susceptible to environmental stress and nutritional deficiencies [2]. These diseases are particularly challenging to manage because the related pathogens can remain in soil for a long time, often causing frequent crop damage in affected areas. Conventional chemical management, often involving pesticides and fungicides, provides temporary relief, but it can have detrimental long-term effects. The overuse of chemical treatments can lead to the development of resistance in pathogens, contamination of soil and water, and harm to non-target organisms, including human beings and beneficial soil microbes [3]. Additionally, such practices also aggravate soil degradation and reduce long-term agricultural productivity.

Pathogen management in agriculture not only affects the agricultural systems and environment but also pressurizes farmers with economic and social burdens, especially in developing countries [4]. For instance, Banana Xanthomonas Wilt (BXW) has caused catastrophic yield losses of up to 60% in Uganda and across Central and Eastern Africa, threatening food security and the livelihoods of millions. The disease caused $2 billion in losses annually and forced some families to stop growing bananas in Uganda, where roughly 30% of farmers depend on them for a living [5]. While eradication efforts encounter resistance because of the high costs and farmers’ reluctance to destroy crops, BXW kills entire plants and renders fields unusable for months. Price hikes to make up for losses have put a strain on low-income consumers, and delayed responses exacerbate the spread of disease. BXW has led to economic vulnerability in regions like the Democratic Republic of the Congo and Rwanda, where bananas account for up to 90% of farmers’ income. This issue is exacerbated by farmers’ reluctance to adopt control measures that are perceived to reduce crop quality [6]. Hence, plant diseases have the potential to destabilize livelihoods, threaten food security, and reduce income, highlighting the urgency for the implementation of sustainable, farmer-friendly, and climate-resilient approaches to disease management.

The disease-suppressive soil provides an effective option for the management of soil-related pathogens and diseases. These soils harbor beneficial microbial communities that naturally suppress the plant pathogen development through direct antagonism, such as the competition for nutrients and space, as well as the production of antibiotics, lytic enzymes, and siderophores that inhibit or kill plant pathogens. These beneficial microbes can also activate plant defense mechanisms through the whole plant, such as induced systemic resistance (ISR) of plants against pathogen infections through different mechanisms, such as the regulation of hormone pathways and defense gene expression [7]. These related beneficial microbes include bacteria, fungi, viruses, protists, nematodes, etc., which are widely present around the world. By promoting disease-suppressive soil development, farmers can reduce over-dependence on harmful chemicals while maintaining healthy soil ecosystems, improve crop flexibility, and enhance sustainable agricultural practices [8]. The primary objective of the review article is to discuss the concepts, history, and mechanisms of disease-suppressive soils (DSS), their relationship to soil health, and the approaches to improve the disease suppression processes in soils. This article is based on the hypothesis that better soil health depends on enhanced physical, chemical, and biological properties, which create an optimal environment for beneficial microbes to thrive and function while simultaneously suppressing the disease-causing organisms.

What is Disease-Suppressive Soil?

**Figure 1:**
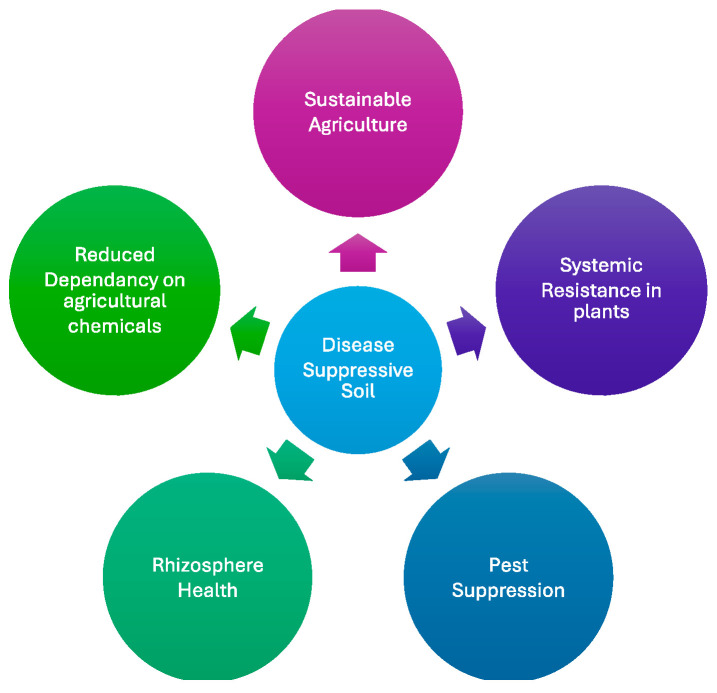
An illustration of the beneficial function of disease-suppressive soils.

The United States Department of Agriculture (USDA) defines soil health as “the continued capacity of soil to function as a vital living ecosystem that sustains plants, animals, and humans”. Disease suppression in soils is crucial as far as soil health is concerned. Disease-suppressive soils are those that naturally inhibit the growth of soil-borne pathogens, reducing disease incidence even without the need for chemical interventions. The phenomenon is recognized as an important component of functional agricultural systems, as it provides an environmentally friendly option for chemical control methods and promotes long-term soil health [9]. The Blenheim soils in Malaysia provide resistance against *Ganoderma basal* stem rot found in oil palms. The shell deposits in these soils lead to the development of specific chitinases and proteases that induce plant defense [10]. Similarly, the morainic soils found in Morens, France, are inherently suppressive to tobacco black root rot caused by *Berkeleyomyces brassicola* [11,12]. Disease-suppressive soil supports sustainable agriculture and provides natural protection against soil-related pathogens. Several studies in the United States have identified potato fields exhibiting specific suppression of *Streptomyces scaleies,* which is often associated with the long-term monoculture [13]. Also, fields with tobacco plantations in China have demonstrated the general suppression of *Fusarium* spp., and flax fields in France have shown general suppression of *Fusarium oxysporum* [14,15,16]. Recent research has strengthened the scientific understanding of this suppression effect. For instance, some studies indicate that the availability of phosphorus (P) can affect the disease suppression of the soil microbiome through plant-microbial interaction [17]. Additionally, the disease-suppressive soil can induce systemic resistance in plants, which enhances their defense mechanisms against diverse pathogens [18]. The role of rhizosphere microorganisms in disease suppression has also been highlighted, with these microorganisms offering effective protection against infections by soil-borne pathogens, including fungi, oomycetes, bacteria, and nematodes [19]. Furthermore, the microbiome of disease-suppressive soils has been linked to the suppression of pests, demonstrating the broader benefits of these soils beyond pathogen control [20] (Figure 1). This review describes the types of disease-suppressive soils, their mechanisms, management practices, and importance in sustainable agriculture.

### 1.1. Types of Disease-Suppressive Soils

There are two primary forms of disease suppression that occur within soils: general and specific suppressiveness. General disease suppressiveness (GDS) refers to a broad capacity of soil to suppress a wide range of soil-borne pathogens, often due to a diverse and robust microbial community that competes with or inhibits the growth of harmful microorganisms. For instance, a well-known GDS example is the protection of wheat plants against the fungal pathogen *Gaeumannomyces graminis var. tritici.* The related disease is called take-all decline (TAD), which occurs worldwide during continuous wheat or barley monoculture. The pathogen inhibition activity is associated with the production of 2,4-diacetylphloroglucinol (2,4-DAPG) with a broad-spectrum antibiotic activity against different pathogens, in this case, the pathogen of *Gaeumannomyces graminis var. tritici.* The 2,4-DAPG was produced by the beneficial bacteria *Pseudomonas* spp. in the soil, which were more abundant after a few years’ monoculture crop practice [21]. In contrast, specific disease suppressiveness (SDS) is more targeted, where the soil’s suppressive properties are effective against certain types of pathogens. For instance, beneficial fungi *Fusarium* spp. in soils can significantly suppress the fungal pathogen to protect tomato (*Solanum lycopersicum*) plants from the development of *Fusarium* wilt disease. The related specific pathogen inhibition activity is associated with the production of peptaibols by beneficial *Fusarium* spp, which act as membrane-active antibiotic compounds [22]. The other example is that the non-pathogenic *Streptomyces rimosus* subsp., which is rich in suppressive soils, can inhibit the *F. graminearum* on wheat ears [23]. Both forms of suppressiveness play a significant role in maintaining plant health, but they function through different ecological mechanisms and are influenced by a variety of factors, including texture, soil organic matter (SOM) content, crop management practices, microbial diversity, soil conditions, and other environmental factors.

The microbial community within the disease-suppressive soil is the major driver of disease and pathogen inhibition. These soils are usually characterized by a high level of microbial diversity, which includes diverse beneficial microorganisms, such as bacteria and fungi. These microorganisms can exert direct antagonistic effects on pathogens through the production of antimicrobial compounds, competition for resources, or the induction of plant defense mechanisms, such as induced systemic resistance (ISR) [24]. In addition, the physical and chemical properties of the soil, such as organic matter (OM) biomass, pH, and soil composition, can contribute to the establishment and maintenance of these beneficial microbial communities for their functions. For example, high SOM content is more likely to harbor a relatively diverse range of microorganisms in the soil with materials that can contribute to disease suppression [25].

Crop management practices, especially crop rotation and organic amendments, play important roles in the development of disease-suppressive soils. For instance, crop rotation helps break the disease cycle by disrupting the life cycles of soil-borne pathogens and promoting the growth of beneficial microbes that may outcompete or inhibit pathogens [24]. Organic amendments, such as manure or fertilizer, further enhance the microbial diversity of soil and promote the growth of beneficial microorganisms, which improves overall disease resistance of the soil. However, the effectiveness of these management practices can be context-dependent, which is affected by factors such as climate, soil type, and pathogen dynamics [26].

Understanding the mechanisms behind disease suppressiveness in soils is important for developing effective disease management strategies. While general disease suppressiveness provides a broad form of disease resistance, specific disease suppressiveness offers targeted control for pathogens, which are of major concern in agriculture. Both forms of suppressiveness are essential to the strategy of reducing the reliance on chemical inputs and promoting sustainable agricultural practices. The ongoing research aims to better understand microbial and environmental factors as well as management approaches that reduce pathogens and diseases, and to translate this knowledge into practical strategies for effectively managing soil pathogens and diseases in diverse agricultural settings.

As research continues to decode the complex interactions within disease-suppressive soils, exploring these natural processes and management approaches can significantly reduce the need for harmful chemical pesticides and contribute to more resilient agricultural systems. Future efforts should focus on characterizing the microbial communities that contribute to both general and specific suppressiveness and developing effective management practices to foster these communities and functions in a sustainable and effective manner [9,24,26].

### 1.2. History of Disease-Suppressive Soils

Disease-suppressive soils, which naturally inhibit the growth of soil-borne pathogens, have long been a subject of interest in plant pathology and soil science. The concept of disease suppression in the soil, although naturally lying in early observations in the disease-free regions, has evolved significantly during the 20th Century. From the report of early anecdotes for the refined studies on microbial ecology, the history of disease-suppressive soil reflects the growing understanding of soil biology, microbial communities, and sustainable agriculture (Table 1). Microbial ecology is the study of how different microorganisms interact with each other, and how they can interact with plants and animals, and with their environment. Microbial ecology is critical to help us study and understand how microbial communities can affect plant growth, development, health, and resilience to biotic and abiotic stresses. Microbes decompose organic matter, and they can further improve plant growth and defense by converting the nutrients, such as nitrogen and phosphorus, into plant-available forms. Beneficial microbes can also compete with pathogens for resources and living space such as around the rhizosphere for survival and further development. In addition, beneficial microbes can produce siderophores to limit iron availability to harmful organisms, so that they limit their growth and development. Additionally, enzymes such as cellulases, phosphatases, and chitinases produced by the beneficial microbes can prevent nutrient release and degrade pathogens. Together, these components function to enhance the suppressiveness of soil-borne pathogens and diseases, which benefits plant health and supports ecosystem stability.

This review explores the historical development of disease-suppressive soils, examining the key milestones, foundational research, and the growing body of evidence supporting the use of disease-suppressive soils in integrated pest management (IPM) and sustainable agricultural practices.

Early Observations and Foundations
  ▪ Pre–1900s

The phenomenon that some soils could suppress disease was first observed in the late 19th and early 20th centuries. Farmers and agronomists in regions with high disease stress, such as those affected by *Fusarium* or *Verticillium* wilt, noted that certain fields always produced more healthy plants, even in the presence of pathogens. These observations, at the same time as anecdotal, laid the foundation for the study of disease-suppressive soils [25].

One of the earliest recognized examples of disease suppression in soils was reported in the 1880s by the Dutch plant pathologist Löffler, who observed that certain soils appeared to suppress the growth of *Fusarium* wilt in tomato plants [27]. However, it was not until the early 20th century that scientists began to focus more systematically on the biological factors responsible for this phenomenon. This groundwork paved the way for major transformations in the mid-1900s, when experimental biology and molecular genetics began reshaping this scientific theme.

Emergence of Modern Disease Suppressiveness Research
  ▪ Mid–20th Century

During the mid-20th century, advances in microbiology and plant pathology led to a more systematic study of disease-suppressive soils. A significant number of studies and observations shaped the theory behind disease suppression. The most significant breakthrough came from the work of Kenneth F. Baker and Robert J. Cook in the 1960s, who formally defined and coined the term “disease-suppressive soils.” They proposed that certain soils had an intrinsic ability to suppress specific soil-borne diseases due to complex microbial interactions [9].

The seminal work of Baker and Cook in the 1960s provided the first clear framework for understanding the mechanisms behind disease suppression. They observed that certain soils were consistently free of specific diseases, even when inoculated with known pathogens. These soils were found to have microbial communities capable of inhibiting pathogens. Baker and Cook proposed that these suppressive effects were mediated by antagonistic microorganisms, which either produced toxic metabolites or directly competed with the pathogens for space and nutrients. This work helped establish the foundation for future research into the microbial ecology of disease-suppressive soils [9].

Expansion of the Concept: General vs. Specific Suppressiveness
  ▪ 1970s–1980s

As research into disease-suppressive soils advanced, it became clear that suppressive properties could be either general or specific in nature. During the 1970s and 1980s, researchers expanded on Baker and Cook’s early work to differentiate between these forms of suppression. The mechanisms behind general suppression were found to involve a broad spectrum of microbial interactions, while specific suppression often relied on the presence of antagonistic organisms, such as *Pseudomonas* or *Trichoderma* species, that targeted specific pathogens [26]. This segmentation increased the complexity of the suppressiveness concept. In order to address such complexities, advanced microbial and molecular techniques emerged in the 1990s.

Advances in Microbial Ecology and Molecular Techniques
  ▪ 1990s–2000s

One of the key breakthroughs during this period was the discovery that the suppressive properties of soils were not just the result of a few dominant microbial species, but rather the outcome of dynamic, multifaceted microbial communities [24]. They reported that culture-independent methods, such as DNA sequencing and the development of metagenomics, allowed researchers to identify microbial communities associated with disease-suppressive soil and identify them in more detail. These advances provided a new insight into microbial players involved in the disease suppression and detected a high level of complexity within the ecosystem of the soil. Another important discovery is that more advanced studies confirmed that some beneficial microbes in disease-suppressive soils, such as *Pseudomonas* and *Bacillus*, can promote plant growth and activate plant-induced systemic resistance (ISR) to confer resistance against different pathogens. This breakthrough not only emphasized the role of beneficial microbes for plant growth but also led researchers to incorporate them into the concept of sustainable agriculture.

Disease-Suppressive Soils and Sustainable Agriculture
  ▪ 2000s–Present

In recent years, the concept of disease-suppressive soils has been increasingly focused on sustainable/regenerative agriculture. Research has shown that some agricultural practices, such as crop rotation, organic amendments, and reduced tillage, are effective in enhancing the disease-suppressive properties of soils [26]. As specifically indicated by Mazzola, these practices promote the establishment of beneficial microbial communities, which in turn help reduce pathogen populations. In his article, Mazzola stated that organic amendments like compost or manure can increase microbial diversity and promote the growth of specific microorganisms that inhibit pathogens. Similarly, long-term crop rotations have been shown to enhance disease suppression, especially when resistant or non-host crops are included, which disrupts the life cycle of soil-borne pathogens [24].

Moreover, the ongoing study of the genetic basis of disease suppression has explored ways to enhance suppressive properties through breeding and biotechnology [24]. Rigorous research is being conducted to identify genetic markers associated with disease resistance in plants and beneficial microbial strains, which could be used to develop crops and soils with enhanced disease-suppressive capabilities while benefiting sustainable agriculture.

## 2. Diverse and Dynamic Mechanisms of the Pathogen Inhibition Caused by Disease-Suppressive Soils

Disease-suppressive soils can inhibit soil-borne pathogens’ infection of host plants through complex interactions among the soil microbiome, plant roots, and the whole environment, which can be associated with diverse and dynamic mechanisms. For instance, global changes, particularly increasing temperatures, can significantly affect the activities of pathogens, plants, beneficial microbes, soils, and their interactions. Rising temperatures and extreme weather can enhance the spread and severity of pathogens such as *Phytophthora* infestans and *Fusarium* species. Under these stressful conditions, the disease-suppressive effects of beneficial soil microbes may be negatively impacted, as their diversity and activity, especially in nutrient cycling, metabolite and enzyme production, and microbial competition, can be reduced. This disruption weakens plant immunity and overall soil resilience. Therefore, managing and preserving soil microbial health is essential for maintaining effective disease suppression in the face of climate change.

### 2.1. General Suppression Mechanisms (GSMs) and Specific Suppression Mechanisms (SSMs)

GSMs involve broad, non-specific microbial activity that suppresses a wide range of soil-borne pathogens through general antagonism and competition, which needs the overall soil microbial community to work together. In contrast, SSMs rely on particular microbial species that actively target specific pathogens using diverse mechanisms, such as the production of specific antimicrobial substances, predation, competition for resources, and stimulation of the host’s immune response.

#### 2.1.1. General Antibiosis (Production of General Antibiotics)

##### General Antibiosis

General antibiosis is a general suppression mechanism where soil microorganisms produce antimicrobial substances that inhibit or kill plant pathogens. This form of suppression is essential for maintaining soil health and preventing the spread of pathogens and diseases. Soil bacteria, such as *Pseudomonas fluorescens* and *Bacillus subtilis*, are well-known for their ability to produce antibiotics like phenazines and cyclic lipopeptides, respectively, which can suppress a large range of fungal and bacterial pathogens. These antimicrobial compounds disrupt pathogen growth by interfering with their cellular processes or by directly causing cell death. The broad-spectrum nature of this type of antibiosis means that it can affect a variety of pathogens, providing a general protective effect for plants growing in the soils [28,29]. One example of general antibiosis has been found in the study conducted by Zhang et al., 2020 [30], where they have discovered the inhibitory action of *Pseudomonas protegens* against *Rhizoctonia solani*, *Pythium ultimum*, *Gaeumannomyces graminis var. tritici*, *Sclerotinia sclerotiorum*, *Fusarium culmorum*, and *F. pseudograminearum*. The related inhibition activities are associated with the production of antimicrobial substances, such as hydrogen cyanide (HCN), pyrrolnitrin, 2,4-Diacetylphloroglucinol (DAPG), pyoluteorin, and phenazines. *Trichoderma* had also been reported to be effective against several pathogens causing different destructive diseases in plants through the production of gliotoxin, peptaibols, viridin, and 6-pentyl-α-pyrone [31].

##### Competition

Competition involves soil microorganisms competing with pathogens for essential resources, such as nutrients and space. The competition can be cooperative, where interactions are mutualistic, and on the other hand, competition can also be negative and leading to direct and indirect exploitative effects [32,33,34]. This competition is a critical general suppression mechanism because it reduces the resources available to all pathogens, thereby limiting their ability to establish and proliferate. For example, beneficial soil fungi *Trichoderma* spp. and bacteria *Bacillus* spp. can outcompete pathogenic fungi and bacteria for nutrients and space to reduce pathogen populations. This competitive exclusion is a non-specific form of suppression that enhances overall soil health and reduces disease incidence. By dominating the soil environment, these beneficial microorganisms create an inhospitable niche for potential pathogens [25,35].

The competition for resources can also work as one of the major GSMs. For example, it has been observed that the competition for nutrients between beneficial microbes and soil-borne pathogens had significant effects on suppressing the population of soil-borne pathogens, thereby protecting the plants [36,37]. It has also been observed that the application of multi-strain biological control agents in the soil has profound effects in disease suppression in soil by enhancing the competition between microbes for nutrients [38].

#### 2.1.2. Specific Suppression Mechanisms (SSMs) Refers to the Targeted Biological Processes in Disease-Suppressive Soils That Inhibit Specific Soil-Borne Pathogens Through Different Mechanisms

##### Specific Antibiosis (Production of Specific Antimicrobial Substances)

There have been many experiments validating the role of specific antimicrobial substances in managing the soil-borne pathogens and diseases. The recent concept of disease-suppressive soil (DSS) gained attention in plant protection by introducing a mechanism by which soil microbes can produce a natural barrier system with a highly concentrated microbial community in the rhizosphere, even in susceptible host plants [39,40]. These microbes can produce narrow-spectrum antibiotics in response to the presence of their target pathogen. For instance, *Pseudomonas fluorescens* strains can produce 2,4-diacetylphloroglucinol (DAPG) to suppress *Gaeumannomyces graminis*, the causal agent of take-all disease in wheat, by disrupting membrane integrity to reduce fungal growth [41]. Another example is the study conducted by Kumari et al. 2021 [42], where *Bacillus* sp. was found to produce iturins and surfactins to confer antagonistic effects specifically against *Sclerotium rolfsii*.

#### Predation

Predation is a SSM in which soil-dwelling predators, such as nematodes, protozoa, or microarthropods, feed on plant pathogens. This interaction is highly specific, with different predators targeting different pathogens. For instance, predatory nematodes like *Hoplolaimus* spp. consume soil-borne fungal pathogens *Fusarium* spp., thereby reducing their population and disease potential. This targeted predation not only decreases pathogen numbers but also disrupts their lifecycle, contributing to long-term disease suppression. The specificity of predation means that each predator-prey interaction can be optimized for controlling different pathogens [43,44]. For example, the positive impact of predation was observed in the study conducted by Erktan et al. (2020) [45], where saprophytic fungi have shown the highest positive effects on soil aggregate formation and stabilization, which is closely associated with the efficient use of particulate organic carbon (POC) in the soil. Predatory nematodes, like Protorhabditis, are shown to have higher impacts on the diversity and stability of soil bacterial communities, leading to the high affinity of C and N metabolism by the microbes [46].

#### Parasitism

Parasitism is another specific suppression mechanism where one organism (the parasite) directly harms or kills another (the pathogen). Parasitic microorganisms, such as certain fungi and bacteria, can infect and destroy plant pathogens. For example, the parasitic fungus *Coniothyrium minitans* targets the sclerotia of the soil-borne fungal pathogen *Sclerotinia sclerotiorum*, leading to a reduction in pathogen populations and disease severity. This specific interaction helps in controlling pathogens by directly targeting and depleting their populations, thus providing effective disease control in the soil [47,48]. Parasitism has also been found to have positive impacts on enhancing N fixation by bacteria while suppressing nematode parasitism in tomato plants [49]. Additionally, root-knot nematode-parasitized tomato plants have been found to harbor a higher population of bacteria belonging to the Rhizobiales, Betaproteobacteriales, and Rhodobacterales, along with their bacterial pathogens, significantly enhancing the expression of the NifH proteins, which are key factors involved in biological nitrogen fixation (BNF) [49]. Cai et al. (2023) [50] reported that the presence of soil microbes enhanced the deleterious effects of the parasite *Cuscuta grovonii* on *Alternanthera philoxeroides*, which indicates that parasitism can alter the influence of native soil microbes on plant growth. Additionally, it has been observed that the presence of certain pathogens, such as *C. campestris*, caused the significant reduction of *Mikania micrantha* population, showing the positive impact of parasitism on plant growth and development [51].

#### Induced Systemic Resistance (ISR) of Plants Against Pathogens Activated by Beneficial Microbes in Soils

Plant growth-promoting microorganisms (PGPM) in the soils, such as bacteria and fungi, play crucial roles in enhancing plant growth, development, and resilience against biotic and abiotic stresses [52]. These microorganisms can improve water and nutrient uptake, directly inhibit pathogens and pests, and activate plant defense mechanisms against biotic and abiotic stressors [53]. Key bacterial genera, such as *Rhizobium*, *Pseudomonas*, and *Bacillus*, facilitate nutrient absorption and stimulate plant defense [54]. Likewise, key fungi, such as arbuscular mycorrhizal fungi (AMF) and Trichoderma, enhance water and nutrient uptake through symbiotic interactions, promoting plant survival in challenging environments [55,56]. PGPM employ diverse mechanisms, including the production of growth-promoting compounds, increased nutrient bioavailability, and improved stress tolerance against biotic and abiotic stresses [57,58,59]. For example, nitrogen-fixing Rhizobium converts atmospheric nitrogen into a plant-available form through symbiosis [60], while potassium-solubilizing bacteria (KSB) release potassium from insoluble minerals, enhancing plant nutrient uptake [61]. Additionally, PGPM produce phytohormones such as auxins and gibberellins to regulate root architecture and further promote plant growth [62].

Plants encounter both biotic (e.g., pathogens, insects) and abiotic (e.g., drought, salinity, extreme temperatures) stresses. Plants can defend themselves against pathogen infections mainly by relying on two primary induced resistance mechanisms: systemic acquired resistance (SAR) and induced systemic resistance (ISR) [63]. Plant systemic resistance is a whole-plant defense response after an initial local stimulus by microbes, so that the uninfected tissues of the plants will be protected against pathogen infections. Both ISR and SAR are plant systemic resistance activated by microbes through the whole plant to confer resistance against different pathogens, which are long-lasting and broad-spectrum resistance. Both SAR and ISR are critical components of the plant immune system, which can be applied in enhancing disease resistance and reducing harmful chemical applications for sustainable agriculture. SAR is generally triggered by pathogens, and ISR is triggered by beneficial microbes. SAR is mediated by salicylic acid (SA) and involves the hypersensitive response (HR), a localized cell death process that prevents pathogen spread [64]. This immune memory enhances plant resistance to future infections. In contrast, ISR is activated by beneficial microbes such as plant growth-promoting rhizobacteria (PGPR) and mycorrhizal fungi in the soil, which is generally regulated by jasmonic acid (JA) and ethylene (ET) [65]. For example, root colonization by *Pseudomonas fluorescens* WCS417r in Arabidopsis triggers ISR via JA/ET signaling, with the MYB72 transcription factor playing a key role [66]. Similarly, *Bacillus megaterium* TRS-4 produces peroxidase, chitinase, and β-1,3-glucanase to suppress fungal pathogens [67]. Plants primed with ISR rapidly generate reactive oxygen species (ROS), antimicrobial compounds, and defense enzymes, strengthening their defenses [68,69]. Bacterial strains, such as *Bacillus amyloliquefaciens GD4a* and *Bacillus proteolyticus OSUB18,* have demonstrated remarkable efficacy in promoting plant growth and suppressing diverse pathogens and diseases through the related mechanisms in Arabidopsis and tomato plants. They achieve pathogen inhibition through ISR and direct antagonism against a wide spectrum of pathogens [2,62,70]. The other example, *Bacillus subtilis* UMAF6639, enhances ROS production in melon plants, reinforcing cell walls against *Podosphaera fusca* infections [71]. Though SAR and ISR function by distinct signaling pathways, they can interact synergistically or antagonistically, depending on the pathogens, hosts, and environmental conditions, providing a coordinated immune response [72]. The intricate interplay between SAR and ISR highlights plants’ remarkable adaptability in responding to environmental stressors, such as pathogen infections. Understanding these mechanisms not only advances knowledge of plant immunity but also fosters the development of sustainable agricultural practices that enhance crop resilience and productivity in the face of global challenges.

### 2.2. Reprogramming of Microbial Community in Soil

The development of disease-suppressive soils involves dynamic reprogramming of the compositions and functions of microbial communities in plants and soils, which help control plant pathogens through various mechanisms. In the case of take-all disease of wheat, caused by the fungal pathogen *Gaeumannomyces tritici*, disease suppression is often linked to specific microbial changes in the bulk soil and rhizosphere. Rhizosphere is the zone of soil that closely surrounds the plant roots and can have extensive interactions with plant roots. For instance, the *Pseudomonas* spp. were more abundant in the bulk soil and rhizosphere under the pathogen pressure, which can produce antifungal compounds like 2,4-diacetylphloroglucinol (DAPG), to inhibit *G. tritici* as mentioned above [73]. Another example is *Bacillus* spp., which can produce lipopeptides and other antimicrobial compounds [74]. Microbial community reprogramming has emerged as a promising approach for enhancing soil health and disease suppressiveness in agricultural systems. Recent advances in synthetic biology, omic techniques, and precision agricultural approaches offer new avenues to modify microbial communities in targeted ways for enhancing the efficacy of disease-suppressive soils.

One of the most significant advances in microbial community reprogramming is the application of synthetic biology to engineer specific microbial traits. Technologies such as CRISPR/Cas9 have enabled precise genetic modifications that enhance beneficial microbial functions, such as root colonization, competitive ability, nutrient cycling, biocontrol activities of pathogens, and other stress resilience. By targeting specific genes responsible for antibiotic production, such as lipopeptides, chitinases, or siderophores, researchers can bolster the pathogen inhibition activities and plant natural defense mechanisms within soil microbial communities [75]. Moreover, synthetic microbial consortia are being developed to perform specialized functions that native communities may not be capable of achieving on their own. For instance, we can utilize synthetic biology to design microbial consortia with optimized metabolic pathways and synergistic responses to plants and microbial signals, so that they can enhance the defense network and final outcomes in the rhizosphere of the plants under different environmental conditions. Therefore, these custom-designed consortia combine species with complementary traits to optimize disease suppression and plant growth promotion [76]. In addition, engineered microbial consortia can enhance nitrogen fixation and pathogen suppression simultaneously, demonstrating their potential in reducing reliance on chemical fertilizers and pesticides [77]. Furthermore, engineered microbial communities can be improved to maintain functionality and sustainability of agricultural systems under climate stress, such as high temperatures or drought/salt stresses, to secure disease suppression in changing environments. These approaches provide efficient tools for us to develop sustainable agriculture through beneficial microbes.

Another key strategy for reprogramming microbial communities lies in the use of organic amendments (OAs) such as compost, biochar, and plant residues. These materials modify the soil environment to favor beneficial microbes, thereby shifting microbial community dynamics [78]. The long-term application of OAs can improve soil suppressiveness against root pathogens, although effects vary depending on the plants and pathosystems [79]. Compost is particularly effective, showing disease suppression in over 50% of cases, while crop residues have more variable effects [80]. OAs can selectively enhance populations of beneficial microbes that suppress pathogens such as *Fusarium*, *Verticillium*, and *Phytophthora*, though they are less effective against soil-borne fungal pathogen *Rhizoctonia solani* [79,80]. The suppressive effect is attributed to the evolution of microbial communities in response to nutrient enrichment, forming functional consortia that exploit available resources and outcompete pathogens [81]. This approach represents a sustainable strategy for biological control of soil-borne diseases in agriculture.

Recent advancements in omics approaches, such as metagenomics, have significantly enhanced our understanding of soil microbial communities, enabling real-time monitoring of their compositions and functions [82,83]. These data-driven approaches allow researchers to assess how management practices influence soil microbiomes, facilitating targeted interventions to promote beneficial microbial groups [84]. Furthermore, the integration of machine learning with omics data aids in predicting the impacts of various soil management strategies on microbial dynamics, optimizing inputs like organic amendments [85]. Meta-omics techniques, including high-throughput sequencing, have emerged as powerful tools for characterizing microbial diversity and functions, providing robust indicators for soil quality assessment and enhancing agricultural sustainability [84]. Overall, these innovations are pivotal for improving soil health and resilience in agricultural systems [83].

Microbial community reprogramming in soil shows great promise in enhancing soil disease suppressiveness and improving crop health without chemical treatments. Organic amendments and beneficial microbes can be combined to modulate the plant-associated microbiome, promoting natural disease suppression [78]. Disease-suppressive compost can enhance soil suppressiveness by introducing beneficial microbiota and stimulating changes in soil microbial communities [86]. Organic farming practices and microbial metabolites contribute to disease-suppressive soils, with certain bacterial strains producing key metabolites that offer mechanistic insights into suppressive phenotypes [87]. While traditional biopesticide approaches using a single strain have limitations, emerging omics tools enable the characterization of microbial consortia in natural systems, allowing for the construction of synthetic microbiomes for effective disease control. Additionally, harnessing indigenous soil microbiomes through specific management strategies presents opportunities for effective disease suppression in agricultural systems [88].

### 2.3. Soil-Driven Mechanisms of Disease Suppression in Soils

Soil’s physical, chemical, and biological parameters play crucial roles in determining the disease-suppressive properties of soils, with their interactions influencing the establishment and activity of beneficial microorganisms that suppress soil-borne pathogens [9]. Baker & Cook (1974) [9] stated that well-structured soils with adequate porosity and aeration provide optimal conditions for microbial growth, allowing beneficial microorganisms to outcompete or antagonize pathogens. They further stated that soil texture also influences water retention and nutrient availability, both of which can impact microbial activity and pathogen survival. Moreover, loam and sandy soils are often more conducive to disease suppression due to their favorable moisture regimes and better root penetration compared to clay-heavy soils.

Mazzola (2002) [26] stated that organic matters, especially from compost or plant and animal debris, act as substrates for microbial populations and enhance microbial diversity, both of which contribute to disease resistance. Authors also mentioned that high OM contents are linked to the increased populations of disease-suppressive microorganisms, such as *Pseudomonas* spp. and *Trichoderma* spp., which produce antibiotics or compete for resources with pathogens. Soil pH is another important factor, as it influences the solubility of nutrients and the microbial community composition [24]. A study conducted by Tan et al., in 2020 [89] for a long-term maize cropping system revealed the Shannon–Weiner index, a measure of bacterial activity, to be inversely correlated with the pH of the soil (r = −0.66, *p* < 0.01). Similarly, a study of 942 soil samples for seven biomes yielded a higher concentration of microbial diversity at a pH of 6–7, which declined when the pH became lower [90]. On the contrary, extreme pH values can inhibit the growth of disease-suppressive microbes and favor pathogen proliferation [24].

Biologically, the diversity and composition of soil microbiomes are central to disease suppressiveness. Microbial communities in soils that are rich in beneficial bacteria, fungi, and actinomycetes can inhibit pathogens through a variety of mechanisms, as mentioned above. For example, *Trichoderma* fungi are well-known for their ability to produce enzymes that break down pathogen cell walls, while certain soil bacteria, such as *Bacillus* spp., produce antimicrobial compounds that suppress destructive soil-borne pathogens like *Fusarium* and *Rhizoctonia* [25]. Furthermore, the presence of mycorrhizal fungi can improve plant health by enhancing nutrient uptake and strengthening the plant’s immune response, contributing to disease suppression. The interactions between these beneficial microbes are often influenced by soil management practices, such as organic amendments, reduced tillage, and crop rotation, which promote microbial diversity and function, further enhancing disease suppression [24].

## 3. Soil Health Parameters Affecting the Activities of Disease Suppression

Soil health is defined as the continued capacity of soil to function as a vital living ecosystem that sustains plants, animals, and humans [91]. One of the most important functions of soil health is its ability to suppress soil-borne diseases, a property that is of increasing interest due to the rise in resistance to synthetic chemicals and the drive for sustainable agricultural practices [92,93]. Several studies have highlighted the interaction between different soil parameters and disease suppression, emphasizing the need for a comprehensive understanding of soil biology, chemistry, and physics to improve disease management strategies [91,94]. The relationship between soil health parameters and disease suppression activities is complex and varies depending on soil type, climate, and agricultural practices employed [95,96]. For instance, soil pH, OM content, microbial diversity, and nutrient availability are some of the primary factors that determine the level of disease suppression [91,92].

### 3.1. Soil pH and Soil Electrical Conductivity (EC)

Soil pH is one of the most studied parameters influencing soil health and disease suppression. Soil pH plays an important role in the nutrient availability, microbial activity, and overall functioning of the plant. Microbial communities are sensitive to pH, which affects vital processes like N fixation, nutrient assimilation, and OM decomposition [97]. Both acidic and alkaline soils can exhibit disease-suppressive properties, but these properties are often attributed to different mechanisms. Studies have shown that beneficial microorganisms, such as *Trichoderma* spp. and *Pseudomonas* spp., thrive in neutral to slightly alkaline soils, where they can effectively suppress pathogens like *Fusarium* and *Rhizoctonia* [92]. These microorganisms often produce antifungal metabolites that inhibit pathogen growth [93]. Some researchers believed that acidic soils could suppress certain pathogens due to the dominance of specific microbial populations that are adapted to lower pH environments by proving the example for *Phytophthora* spp. On the contrary, Mitsuboshi et al., 2022 [98] reported a partial regression coefficient of −6.95 (*p* < 0.01) for the soils with an incidence of spinach wilt by *Fusarium oxysporum*, suggesting the associations of lower pH with higher disease incidences. The microbial community’s structure is directly impacted by pH, as it affects the solubility and bioavailability of essential nutrients like N, P, and K. At suboptimal pH levels, certain beneficial microbes are less effective at nutrient cycling and pathogen suppression [94]. Thus, maintaining a balanced pH within an optimal range (generally 6–7 for most crops) is critical for promoting disease suppression.

Soil electrical conductivity (EC) indicates the ability of soil water to carry electrical current (USDA). EC reflects the concentration of soluble salts in the soil, which directly impacts the ionic composition of the rhizosphere and the soil microbial community. When the EC levels are higher in soils, uptake of water by soils is affected due to higher osmotic pressure, which compromises the plant’s growth. It also affects soil microbial balance by reducing the activity of beneficial microbes and favoring the activities of pathogenic microbes [99,100]. High EC levels are often associated with excessive fertilization or poor drainage, which can lead to soil salinization, which stresses plants and soil organisms [101]. For the related study, they discussed how high salt concentrations can reduce microbial diversity and favor the growth of certain pathogens by reducing disease suppression. Conversely, low EC levels may also impair nutrient availability, limiting plant growth and disease resistance. However, studies have shown that certain soil-borne pathogens, including those responsible for damping-off diseases, are less prevalent in soils with moderate EC levels, which might be due to the inhibitory effects of salts on pathogen growth [94]. Therefore, while extreme EC levels can be detrimental, moderate EC levels can have a beneficial role in disease suppression by maintaining a balanced microbial community.

### 3.2. Soil Temperature and Moisture

Temperature is another critical factor influencing soil health and disease suppressiveness. The thermal regime of the soil affects microbial activity, pathogen survival, and the dynamics of soil-borne diseases [91]. Larkin stated that soil-borne pathogens tend to thrive in certain temperature ranges, and extreme temperatures can disrupt the pathogen lifecycle. He further discussed how certain *Fusarium* species could show increased pathogenicity in warmer soils, while others, like *Verticillium*, are more problematic in soils with lower temperatures.

Beneficial microorganisms tend to inhibit pathogens at a temperature of around 20–30 °C due to their faster growth rates and production of antimicrobial compounds [96]. However, Sagova Mareckova and fellow researchers also stated that elevated temperatures may lead to increased pathogen virulence, especially under water-stressed conditions, altering the balance between beneficial microbes and pathogens. This underlines the importance of integrated soil management practices that regulate temperature and moisture conditions to enhance disease suppression.

Soil moisture is intrinsically linked to disease suppressiveness because it directly affects microbial activity and pathogen behavior. Both excess moisture and drought can disrupt the disease-suppressive properties of soils [102]. Some researchers concluded that high moisture levels favor the proliferation of waterborne pathogens like *Pythium* and *Phytophthora*, while low moisture levels suppress plants and soil organisms, reducing microbial activity and disease resistance. According to their research, soil moisture content influences microbial diversity by selecting for groups that are either adapted to anaerobic conditions or those that thrive in dry environments. Yet, Cardoso et al., (2013) [93] reported that well-drained soils with moderate moisture levels tend to have a higher microbial biomass, which is essential for disease suppression. Therefore, maintaining proper moisture regimes through irrigation management is essential for sustaining soil health and promoting disease suppression.

### 3.3. Soil Clay Content and Soil Aggregation

The texture of the soil, particularly its clay content and the aggregation of soil particles, plays a significant role in disease suppression. Jayaramanet al. (2021) [94] stated that clay-rich soils generally exhibit higher cation exchange capacity (CEC), which can enhance nutrient availability and microbial activity. Smectite and vermiculite possess a CEC, which contributes to their significant buffering ability. This characteristic may be linked to the presence of numerous charged elements, such as organic matter, thereby enhancing their ability to suppress diseases like *Fusarium* wilt (*Fusarium oxysporum f.* sp. *cubense* (Foc)) in bananas [103]. Similarly, Persson and Olsson (2000) [104] identified a strong positive correlation between the suppression of Aphanomyces root rot and the ratio of vermiculite–smectite to illite–kaolinite, along with increased clay content, soil pH, and soil Ca levels. Jayaraman et al. (2021) [94] further stated that clay particles provide more surface area for microbial processes and nutrient retention, facilitating a susceptible environment for beneficial microbes that can suppress pathogens.

Soil aggregation is also crucial in promoting disease suppression. Aggregates help protect beneficial microbes from environmental stresses and improve soil structure, thereby enhancing soil aeration and water infiltration [96]. They further discussed the importance of well-aggregated soils in supporting the growth of beneficial fungi and bacteria, contributing to disease resistance by directly antagonizing soil-borne pathogens. In contrast, poorly aggregated soils may have reduced microbial activity and increased vulnerability to pathogens due to less efficient nutrient cycling and lower microbial diversity.

### 3.4. Soil Carbon and Nitrogen Pools and Stocks

The SOC and N pools are fundamental components of soil health and play an essential role in enhancing disease-suppressive properties [96]. Mareckova et al. (2023) [96] discussed the role of SOC as a primary energy source for soil microorganisms and how it impacts the diversity of soil microflora and microfauna. Soils rich in OM support a diverse microbial community, including antagonistic bacteria and fungi that suppress plant diseases [95]. A study of the relationship between SOC fractions and fungi stasis conducted on the tillage systems and crop sequences in a boreal agroecosystem by Palojarvi et al., 2020 [105] revealed the particulate organic matter carbon (POM-C), permanganate oxidizable carbon (POX-C), and microbial biomass carbon (Cmic) to be significantly higher under reduced-tillage practices. The correlation was identified to be the strongest with the labile carbon POM-C (*r* = −0.40, *p* = 0.05) and moderate with the Cmic (*r* = −0.34, *p* = 0.06). The growth of *Fusarium culmorum* was found to be inversely related to these labile C fractions, indicating an increase in labile C supporting disease suppression by promoting beneficial microbial communities.

N availability also influences disease suppression, as it affects plant growth and the microbial community. High N levels can promote the growth of certain pathogens by enhancing plant susceptibility, as pathogens like *Fusarium* and Rhizoctonia thrive on nitrogen-rich plants [101]. However, N also supports the growth of beneficial microorganisms that can suppress pathogens. Therefore, the balance of N in the soil is crucial for disease suppression, and management practices, such as crop rotation and organic amendments, can help maintain optimal N levels.

The studies highlighted that soil health parameters do not act in isolation; rather, they interact in complex ways to influence disease suppression. For example, a balanced soil pH, optimal moisture, and appropriate temperature conditions are all necessary to maintain a thriving microbial community that can effectively suppress soil-borne pathogens. Organic amendments, including cover crops and composts, contribute to increasing SOM contents, thereby improving microbial activity and disease suppression [102]. However, the challenge lies in understanding the dynamic interactions between these parameters and their combined effect on disease suppression. For instance, soil amendments that increase OM may also alter pH or EC, leading to shifts in microbial community composition. Therefore, integrated management practices that consider all these factors are necessary for sustainable disease management [92].

Disease-suppressive soils play critical roles in maintaining soil health and improving C and N stocks, which are essential for sustainable agricultural productivity. These soils possess microbial communities that naturally inhibit soil-borne pathogens, reducing the need for chemical interventions while enhancing SOM accumulation [106]. The suppression of diseases like *Fusarium* wilt and Rhizoctonia root rot is linked to specific microbial interactions that contribute to greater stability in soil C and N cycles [107].

Soil microbial diversity and composition significantly influence disease suppression, leading to increased SOC sequestration. Beneficial microbes, particularly members of the phyla Actinobacteria and Proteobacteria, contribute to the decomposition of OM, thereby stabilizing soil C pools [80]. The presence of pathogen-suppressing microbes such as Pseudomonas and Bacillus spp. enhances rhizodeposition, which further facilitates C inputs into the soil system [108,109]. Suppressive soils tend to have higher microbial biomass and enzyme activities, which accelerate nutrient cycling and contribute to C sequestration [110].

N cycling in disease-suppressive soils is also enhanced through microbial-mediated processes, particularly N fixation and mineralization. Symbiotic and free-living N-fixing bacteria, such as *Rhizobia* and *Azospirillum*, thrive in biologically active suppressive soils, improving plant-available nitrogen [111]. Additionally, disease suppression mechanisms often coincide with an increase in mycorrhizal associations, which improve N uptake efficiency in plants, reducing dependency on synthetic fertilizers [112]. Higher microbial respiration rates in these soils indicate an active turnover of OM, ensuring a steady release of N while minimizing leaching losses [113].

Management practices that promote disease-suppressive soils, such as organic amendments and crop rotations, have a direct impact on soil C and N stocks. Compost and biochar applications enhance microbial diversity and suppress pathogenic fungi, leading to improved soil aggregation and increased C sequestration potential [114]. Cover cropping and intercropping systems further enrich soil microbial networks, sustaining long-term C inputs and stabilizing N availability [115]. No-till and reduced-tillage practices also encourage the persistence of beneficial microbes, thereby fostering disease suppression while reducing soil C losses [41].

The role of disease-suppressive soils in mitigating climate change through C sequestration is gaining attention. By fostering microbial communities that regulate greenhouse gas emissions, these soils help maintain a balance between C storage and release [116]. Research suggests that increasing disease suppression potential in agricultural soils could significantly enhance soil resilience to climate variability, further improving N use efficiency and reducing N_2_O emissions [38]. Understanding the intricate relationships between disease suppression, soil microbial ecology, and nutrient cycling is crucial for developing sustainable agricultural practices that simultaneously promote plant health and ecosystem stability [107].

### 3.5. Soil Respiration

The USDA describes soil respiration as the release of CO_2_ from the soil surface. Soil respiration is the outcome of the microbial breakdown of soil organic matter. It reflects the capacity of soil to support different forms of life, including crops, soil microorganisms, and soil microflora and microfauna. Soils with a higher respiration rate are an indication of ground for diverse microbial activity. A higher population of soil microbes plays a key role in limiting soil-borne pathogens by competing for resources, producing antimicrobial substances, or preying on harmful organisms. A well-balanced microbial ecosystem supports general suppression by maintaining ecological stability and making it difficult for pathogens to establish. Additionally, specific suppressiveness may be linked to the production of anti-fungal compounds that might induce systemic resistance in plants. Therefore, monitoring CO_2_ evolution serves as a crucial indicator of soil biological activity [41,94].

## 4. Disease-Suppressive Soils—Management Trends

The management of disease-suppressive soils is crucial for sustainable agriculture, as soil-borne pathogens significantly impact crop productivity and food security [96]. Understanding the interactions between root exudates and rhizosphere microbial communities is crucial for managing soil-borne diseases. Root exudates are compounds produced by plant roots, which can be released to the surrounding soil and affect the soil function, including microbial activities. These compounds include sugars, amino acids, vitamins, enzymes, organic acids, and secondary metabolites, which can benefit plants for growth, development, and defense against biotic and abiotic stresses. For instance, a comparative analysis of root exudates and rhizosphere soil bacterial assembly between tomatoes and peppers infected by *Ralstonia* spp. reveals how different host plants influence the rhizosphere environment and disease outcomes [100]. This highlights the need for plant-specific soil management strategies to maximize disease suppression. Disease-suppressive soils naturally inhibit plant diseases through complex interactions among soil microbiota, environmental conditions, and host plants [102]. Effective management strategies can include agricultural practices, such as crop rotation, reduced tillage, cover cropping, biochar application, organic amendments (e.g., Anaerobic soil disinfestation), biocontrol agents, microbiome engineering, and nanoparticle applications [117]. These approaches enhance soil microbial diversity and resilience, reducing pathogen growth and enhancing long-term soil health. Developing sustainable disease-suppressive soils is essential for mitigating agricultural losses while minimizing chemical pesticide use.

Crop rotation is one of the most effective practices for disease management [118]. By alternating crops that are not susceptible to the same pathogens, crop rotation disrupts the life cycle of soil-borne pathogens, reducing their buildup in the soil. Bever et al. (2012) [119] also reported that rotating crops with non-host or resistant crops can lower pathogen populations and promote the growth of beneficial microorganisms that antagonize pathogens. Fravel et al. (2003) [120] demonstrated that this practice could help to maintain a balance in the microbial community, preventing the dominance of pathogenic species. He further stated that rotating legumes with cereal crops can reduce *Fusarium* wilt and *Verticillium* wilt, as these pathogens are often crop-specific. A long-term potato rotation study by Larkin RP (2013) [121] identified a drop in the soil-borne disease of about 20–40% and a further 8–13% reduction (a combined reduction of 30–50%) in disease severity was observed when the crop rotation was paired with winter rye (fall cover crop).

Reduced tillage is another agronomic practice that helps suppress soil-borne diseases by maintaining soil structure and a stable soil ecosystem [110]. Larkin and fellow researchers have stated that tillage disrupts soil microbial communities, reducing the populations of beneficial organisms, which in turn makes the soil environment conducive for pathogen growth. As per them, minimizing soil disturbance by reduced tillage preserves the integrity of soil aggregates, improving habitat conditions for beneficial microbes while reducing pathogen survival. This practice also promotes the buildup of OM and encourages the establishment of symbiotic relationships between plants and soil microorganisms, which can enhance plant resistance to pathogens and diseases.

Cover cropping is widely used to suppress soil-borne pathogens and improve soil health. Cover crops, such as mustard, radish, and rye, can reduce pathogen populations by releasing biocidal compounds, improving soil structure, and promoting microbial activity [122]. They reported that certain cover crops from the Brassicaceae family, such as mustard, can produce glucosinolates, which have direct toxic effects on soil-borne pathogens like *Pythium* spp. and *Rhizoctonia* spp. In potato rotations in Maine, the use of Brassica green manures led to a notable reduction in diseases, with black scurf, common scab, and powdery scab being effectively reduced in 70%, 41%, and 46% of the trials, respectively [121]. Additionally, an increase in yield was observed in 51% of the trials. This suggests that cover crops help reduce soil erosion and nutrient leaching, creating a more stable environment for beneficial microorganisms that contribute to disease suppression.

In addition to the traditional practices as mentioned above, there are further developed approaches to manage the disease-suppressive soils. For instance, biochar, a C-rich product derived from the pyrolysis of organic materials, plays a crucial role in improving soil health and suppressing plant diseases. Its alkaline pH and porous structure create an environment conducive for beneficial microorganisms to grow, enhancing nutrient availability [123]. They also stated that biochar application is found to effectively manage soil-borne pathogens by inducing systemic resistance in plants and improving soil physical, chemical, and biological attributes. Organic amendments, such as composts and manures, also contribute to disease suppression by enhancing soil microbial diversity and activity [102]. As per them, suppressive organic amendments improve soil quality and plant health by enhancing beneficial microbial communities that inhibit pathogens. In a study by Termorshuizen et al. (2006) [124], a diverse group of researchers from multiple countries assessed various composts against different pathogens and diseases. Out of 120 bioassays involving 18 composts and 7 pathosystems, slightly more than half (54%) demonstrated significant disease suppression. Meanwhile, 43% showed no impact, and only 3% led to an increase in disease, with the effects differing depending on the pathogen. According to Bonanomi et al. (2007) [80], the use of organic wastes, including uncomposted manures and industrial by-products, such as bone meal, fish, and paper mill residues, can lead to disease suppression in over half of the trials, while less than 12% of the trials showed an increase in disease. Additionally, Janvier et al. (2007) [95] discussed that organic amendments act as indicators and help manage soil-borne diseases by promoting soil health and microbial balance.

Biological control methods are also critical in soil-borne disease management by relying on the natural enemies of pathogens. For example, Trichoderma and Pseudomonas are effective biocontrol agents against phytopathogens, utilizing various mechanisms to suppress diseases and enhance plant health [125]. Trichoderma can compete for nutrients, produce antifungal metabolites, and induce resistance in plants [126]. Additionally, arbuscular mycorrhizal fungi (AMF) contribute to plant growth not only by enhancing nutrient uptake but also by improving soil aggregation, plant water relations, and disease tolerance, effects that are often as significant as their role in N and P acquisition [127]. Lan et al. (2024) [128] revealed that antagonistic bacteria exhibit biological control against potato black scurf disease caused by *Rhizoctonia solani*, supporting the use of beneficial microbes in disease suppression in soils. Additionally, Yağmur et al. (2024) [129] discovered that the combined application of AMF and *T. harzianum* significantly reduced *Fusarium* basal rot in onions, highlighting the great potential of biocontrol agents in disease management. The deliberate introduction of beneficial microbial communities through targeted soil management practices has been shown to enhance soil suppressiveness against a broad range of plant pathogens, which can promote microbial diversity and foster antagonistic interactions that inhibit pathogen proliferation [117]. Rhizosphere and root microbiome manipulation has gained attention as a strategy to control soil-borne diseases. Studies show that modifying the soil microbiome through targeted microbial inoculation or organic amendments leads to enhanced pathogen suppression in soils [96].

Recent advances in microbial biotechnology provide promising strategies to exploit disease-suppressive soils for agriculture, as mentioned above. High-throughput sequencing and meta-omics approaches allow the detailed analysis of soil microbiomes, enabling the identification of microbial groups associated with disease suppressiveness [130]. Prebiotics in combination with synthetic microbial communities are promising tools for enhancing plant health and disease resistance [131]. Next-generation sequencing, synthetic biology, and CRISPR-Cas9 form the triangle of emerging technology driving innovation in developing targeted microbial inoculants and engineered microorganisms with premium traits [132]. Machine learning models can accurately identify disease-suppressive soils and potential beneficial bacteria, including Firmicutes and Actinobacteria, which have been shown to activate plant immune responses [133].

The other innovative approaches, such as the application of biologically synthesized nanoparticles and anaerobic soil disinfestation (ASD), have emerged in disease management. Silver nanoparticles synthesized using biological methods effectively suppress root rot fungal diseases in common beans, proving to be a unique strategy for disease control [134]. Anaerobic soil disinfestation (ASD) is another effective method for controlling soil-borne pathogens [135]. They discussed that ASD could enhance soil microbial activity by creating anaerobic conditions that suppress plant pathogens in high-tunnel organic baby leaf lettuce production systems. This technique reduces pathogen viability while improving soil structure and fertility. Vermicompost has been recognized for its dual role in plant growth promotion and disease suppression [136]. They suggested that vermicompost application significantly suppresses damping-off disease in potted vegetable soybean, demonstrating its effectiveness in managing soil-borne pathogens. The microbial richness in vermicompost contributes to pathogen inhibition and overall soil health improvement. Similarly, the use of potassium phosphite can enhance the suppressive capacity of the soil microbiome against pathogens like *Ralstonia solanacearum* in tomatoes [137]. Disease-suppressive soils also benefit from integrated soil fertility management (ISFM) practices. Combining organic amendments with microbial inoculants enhances soil suppressiveness against multiple pathogens [118]. This integrative approach ensures long-term soil health and reduces dependency on chemical pesticides.

**Figure 2:**
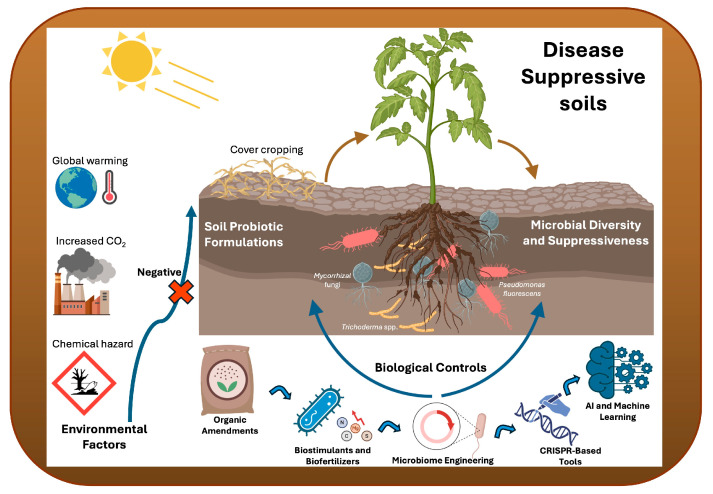
Multifaceted approaches for the development of disease-suppressive soils.

Overall, the development of disease-suppressive soils requires multifaceted approaches (Figure 2). For instance, incorporating biochar application, organic amendments, biocontrol agents, strategic crop rotation, omics approaches, and other innovative techniques is critical. Integrated management practices mitigate soil-borne pathogen impacts while promoting sustainable agricultural productivity by leveraging the natural disease-suppressive capacities of soils. Although the management practices have been developed fast, there are a few challenges to be overcome, including soil ecosystem variability, production costs, and regulatory complexities. The integration of different practices into sustainable agricultural practices could thus contribute to higher productivity with a corresponding environmental benefit in the future [132].

## 5. Limitations of Disease-Suppressive Soils

Disease-suppressive soils, while highly beneficial for agriculture, face several limitations that affect their predictability, establishment, and general understanding. The incidences of disease-suppressive soils are difficult to predict, and the mechanisms driving the establishment of these soils are often poorly characterized. Researchers have reported a high variation of disease suppressiveness in field studies despite having systemic evaluations [26,96,138,139,140]. Often, suppressive soils and their microbiomes are well adapted to the prevailing climatic conditions of a specific region, which indicates the suppressiveness to be local [16,141]. Moreover, the heterogeneity of host plants, management strategies, soil type, and other factors under different experimental settings and variable sites has posed difficulties in the identification of consensus patterns of global microbiomes in the suppressive soils [96,142]. Assessing microbial diversity as an indicator of disease-suppressive soils is yet to be determined, as studies have reported opposing links between suppressiveness and diversity metrics. The prokaryotic patterns that distinguish suppressive patterns from conducive ones are rare, and bioinformatic analyses provide variable results due to the differences in methods and assumptions. Moreover, high-throughput datasets often lack targeted information, making it difficult to fully understand soil functionality [96].

Measures to introduce antagonistic microbial strains into soil fail to last longer because of destruction from agricultural practices like tillage, excessive agro-chemical application, irrigation, and competition from the existing microorganisms [14,143]. Moreover, a lack of comprehensive understanding of the roles of soil organisms in soil functionality hinders the complete benefits of disease-suppressive soils [96].

## 6. Conclusions and Future Insights

**Figure 3:**
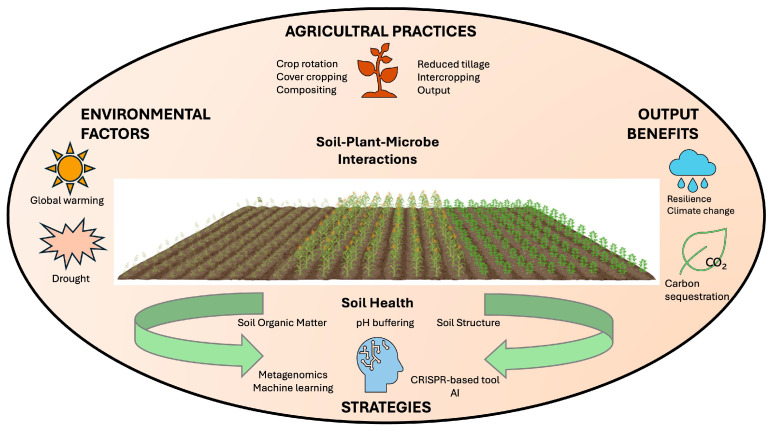
Overview of the history, mechanisms, factors, current status, and future prospects of disease-suppressive soils in agriculture.

The study of disease-suppressive soils holds significant promise for sustainable agriculture, especially in the fields of plant and soil health as well as climate change (Figure 3). Disease-suppressive soils are characterized by their inherent ability to protect plants from soil-borne pathogens through complex interactions within the soil microbiome [107]. Recent advancements in next-generation sequencing and omics approaches have deepened our understanding of these microbiomes, revealing how indigenous microbial communities can reduce disease incidence even in the presence of pathogens and susceptible hosts. Climate change poses additional challenges to soil health by altering environmental conditions that can exacerbate pathogen prevalence and virulence. Rising temperatures, altered precipitation patterns, and increased carbon dioxide levels are driving shifts in the distribution and behavior of plant pathogens, which complicates disease management strategies [144]. Moreover, climate-induced stressors such as heat and drought can weaken plant defenses, making them more susceptible to pathogens [145].

In this context, the future scope of studying disease-suppressive soils is multifaceted. Research is increasingly focusing on identifying specific microbial taxa and assemblages responsible for disease suppression, with the aim of developing microbiome-based biocontrol strategies [24]. Understanding the mechanisms by which these beneficial microorganisms inhibit pathogens can lead to the development of natural and sustainable disease management practices.

Additionally, there is a growing interest in how agricultural practices can be optimized to promote disease suppressiveness. Practices that enhance soil organic matter, maintain biodiversity, and avoid overuse of chemicals are being explored for their potential to promote beneficial microbial communities [146]. Such practices not only contribute to disease suppression but also improve overall soil health, making agroecosystems more resilient to the impacts of climate change. Suppressive soils exhibit distinct chemical and biological characteristics that contribute to pathogen inhibition. Comparative studies on root exudates and rhizosphere microbial assembly provide valuable insights into how soil suppressiveness is mediated through plant-microbe interactions [100]. Understanding these detailed mechanisms can help in designing effective disease management strategies tailored to specific crops and soil types.

Furthermore, integrating the study of disease-suppressive soils with climate change research is crucial. Examining how changing climatic conditions affect the efficacy of disease-suppressive soils will be essential for developing adaptive management strategies. For instance, understanding how temperature and moisture fluctuations influence microbial community dynamics can inform practices that bolster disease suppression under varying environmental conditions [147].

In conclusion, the future scope of studying disease-suppressive soils is expansive and holds the potential to revolutionize sustainable agriculture. By elucidating the complex interactions within soil microbiomes and understanding their responses to climate change, researchers can develop innovative strategies to manage plant diseases naturally. This holistic approach not only addresses the immediate challenges posed by pathogens but also contributes to the long-term health, resilience, and sustainability of agricultural systems.

## Figures and Tables

**Table 1:** History of the development of the understanding and applications of disease-suppressive soils.

History of Disease-Suppressive Soil			
Sl. No.	Year	Advancements	Outcomes
1.	Pre–1900s	Early observations regarding the suppression of *Fusarium* wilt in tomato in Germany	Foundational work that led to the emergence of disease-suppressive soils
2.	Mid–20th Century	Disease-suppressive soils coined by Kenneth F. Baker and Robert J. Cook	The mechanism behind disease-suppressive soils was explored and paved the way for the study of microbial ecology
3.	1970s–1980s	General and specific disease-suppressive soil	The complexity in understanding the suppressive mechanisms led to advanced molecular research.
4.	1990s–2000s	Evolution of DNA sequencing and metagenomic approaches in disease-suppressive soil research. The ISR mechanism was further discovered and studied. The role of beneficial microbes was acknowledged.	The evolution of advanced DNA sequencing and the acknowledgement of beneficial microbes led to the incorporation of these concepts into sustainable agriculture.
5	2000s–Present	Using the concept of disease-suppressive soils to promote sustainable agriculture by decreasing the dependence on agricultural chemicals. Identification of genetic markers associated with disease resistance in plants and beneficial microbial strains.	This approach paves the way for resilient agro-ecosystems and region-specific soil health strategies.

## Data Availability

No new data sets were analyzed for the purpose of this paper.

## Abbreviations

The following abbreviations are used in this manuscript:

AMF	Arbuscular Mycorrhizal Fungi
ASD	Anaerobic Soil Disinfestation
BNF	Biological Nitrogen Fixation
C	Carbon
CEC	Cation Exchange Capacity
Cmic	Microbial Biomass Carbon
DAPG	2,4-Diacetylphoroglucinol
DSS	Disease-Suppressive Soils
EC	Electrical Conductivity
ET	Ethylene
FoC	*Fusarium oxysporum* sp. *cubense*
GDS	General Disease Suppression
GSMs	General Suppression Mechanisms
HCN	Hydrogen Cyanide
HR	Hypersensitive Response
IPM	Integrated Pest Management
ISFM	Integrated Soil Fertility Management
ISR	Induced Systemic Resistance
JA	Jasmonic Acid
KSB	Potassium-Solubilizing Bacteria
N	Nitrogen
OAS	Organic Amendments
OM	Organic Matter
PGPM	Plant Growth-Promoting Microorganisms
PGPR	Plant Growth-Promoting Rhizobacteria
POC	Particulate Organic Matter
POM-C	Particulate Organic Matter Carbon
POX-C	Permanganate Oxidizable Carbon
ROS	Reactive Oxygen Species
SA	Salicylic Acid
SAR	Systemic Acquired Resistance
SDS	Specific Disease Suppression
SOM	Soil Organic Matter
SSMs	Specific Suppression Mechanisms
USDA	The United States Department of Agriculture
